# Precise radiation, better airway preservation: vocal cord-only image-guided intensity-modulated radiation therapy for early-stage glottic cancer

**DOI:** 10.3389/fonc.2026.1847970

**Published:** 2026-05-20

**Authors:** Issa Mohamad, Ibrahim Alotain, Shatha Abu Taha, Mohammad Mukahal, Ayat Taqash, Mohammad Alsmairat, Abdulla Alzibdeh, Mohammad Berawi, Lina Wahbeh, Renda AlHabib, Akram Al-Ibraheem, Hikmat Abdel-Razeq, Fawzi Abuhijla, Ramiz Abu-Hijlih, Omar Al Saraireh, Ali Hosni

**Affiliations:** 1 Department of Radiation Oncology, King Hussein Cancer Center, Jordan, Jordan; 2 Department of Radiation Oncology, King Fahad Specialist, Dammam, Saudi Arabia; 3 Department of Biostatistics, King Hussein Cancer Center, Amman, Jordan; 4 Department of Nuclear Medicine, King Hussein Cancer Center, Amman, Jordan; 5 Department of Internal Medicine, King Hussein Cancer Center, Amman, Jordan; 6 Department of Surgical Oncology, King Hussein Cancer Center, Amman, Jordan; 7 Radiation Medicine Program, Princess Margaret Cancer Center, University Health network, University of Toronto, Toronto, ON, Canada

**Keywords:** laryngeal cancer, local failure, tracheostomy rate, vocal cord-only radiotherapy, whole larynx radiotherapy

## Abstract

**Purpose:**

Radiotherapy (RT) achieves excellent local control (LC) in early-stage glottic cancer (ESGC); however, treatment-related edema or necrosis may necessitate tracheostomy and adversely affect quality of life. We compared tracheostomy rates and oncologic outcomes between whole-larynx radiotherapy (WLRT) and vocal cord–only radiotherapy (VC-RT) in patients with ESGC.

**Methods:**

We retrospectively analyzed 247 patients with Tis–T2 glottic cancer treated with definitive RT (2007–2023) at two Middle Eastern centers. Patients received WLRT (n = 166) or VC-RT (n = 81). The primary endpoint was a tracheostomy rate (TR) ≥6 months after RT for non-recurrent airway compromise. Secondary endpoints included 3-year local failure (LF) and overall survival (OS). TR and LF rates were analyzed using cumulative incidence with death as a competing risk, whereas OS was estimated using the Kaplan–Meier method.

**Results:**

The median age was 59 years; 97.6% of patients were men and 78.9% were smokers. The median follow-up among surviving patients was 38.2 months (range 1.6–194 months). The median potential follow-up for the entire cohort, estimated by the reverse Kaplan–Meier method, was 45.3 months. VC-RT patients more frequently had cT2 disease (22.2% vs. 5.4%, p < 0.001) and received accelerated fractionation (19.8% vs. 7.2%, p = 0.0069). Overall, 20 of 247 patients (8%) required tracheostomy without recurrence. Edema-related tracheostomy occurred exclusively in WLRT patients (14.2% vs. 0% with VC-RT; p < 0.001). Tracheostomy was reversed in 8/20 patients (40%) after a median of 13.4 months. The 3-year LF rates were 7.6% with VC-RT and 11.6% with WLRT (p = 0.18), and OS was 93.3% and 89.5% (p = 0.16), respectively.

**Conclusions:**

VC-RT reduces the risk of tracheostomy without compromising oncologic outcomes.

## Introduction

Early-stage glottic cancer (ESGC; Tis–T2) is among the most curable head and neck cancers (1). Radiation therapy (RT) achieves excellent local control while preserving laryngeal function and voice quality (2). Definitive RT remains a standard treatment option alongside transoral laser microsurgery (3), with reported local control (LC) rates exceeding 85–90% for cT1 disease and 70–85% for cT2 disease (4–6).

Historically, RT for ESGC has targeted the entire larynx, a technique known as whole-larynx radiotherapy (WLRT) (6). This approach originated during the era of two- and three-dimensional conventional radiotherapy (2D/3DCRT) planning to account for uncertainties in tumor localization and motion (6). However, WLRT exposes uninvolved laryngeal structures including the supraglottic and subglottic regions, arytenoids, and surrounding soft tissues to substantial radiation doses (7). Consequently, patients may experience treatment-related toxicities such as laryngeal edema, fibrosis, cartilage necrosis, hypothyroidism, and airway compromise (8). Carotid artery irradiation has also been associated with increased cerebrovascular events (9, 10).

Advances in imaging, immobilization, and intensity-modulated radiotherapy (IMRT) now enable more precise targeting of glottic tumors while sparing adjacent normal tissues (11). In this context, the international consensus guidelines by Vincent Grégoire et al. (12) provide a robust framework for clinical target volume (CTV-P) delineation across head and neck subsites, including ESGC, emphasizing the importance of anatomical compartmentalization and patterns of microscopic spread in guiding safe target reduction. Reducing unnecessary radiation to uninvolved laryngeal structures may improve functional outcomes without compromising tumor control. Vocal cord–only radiotherapy (VC-RT) has therefore emerged as a strategy to reduce radiation exposure to uninvolved tissues while maintaining oncologic outcomes in selected cTis–T2 glottic cancers (13, 14). However, concerns remain regarding the potential for geographic miss and the limited data comparing functional complications, particularly tracheostomy, between VC-RT (15) and conventional WLRT (11, 16).

In this multi-institutional study, we compared tracheostomy rates and oncologic outcomes between WLRT and VC-RT in patients with ESGC treated at two centers in the Middle East.

## Materials and methods

### Study population

This retrospective study included patients with ESGC treated with curative-intent RT at two institutions: the Department of Radiation Oncology at King Hussein Cancer Center (KHCC), Amman, Jordan, and the Department of Radiation Oncology, King Fahad Specialist Hospital, Dammam, Saudi Arabia, between January 2007 and October 2023. Institutional review board (IRB) approval was obtained on October 24, 2024 (IRB No. 24 KHCC 221).

Eligible patients were ≥18 years old with cTis–T2 glottic cancer, treated with either WLRT or VC-RT, and staged according to the American Joint Committee on Cancer (AJCC), 8th edition criteria (17). Exclusion criteria included prior head and neck RT or treated with transoral laser microsurgery, total RT dose <50 Gy, Eastern Cooperative Oncology Group (ECOG) performance status (PS) >2, and the presence of metastatic disease or synchronous malignancies.

### Diagnostic evaluation

All patients underwent multidisciplinary evaluation, including a detailed medical history, comprehensive physical examination with fiberoptic nasopharyngolaryngoscopy, direct laryngoscopy with histologic confirmation of malignancy, and imaging with contrasted CT of the head and neck and chest. PET/CT was obtained when clinically indicated. Pretreatment assessments also included dental, nutritional, and speech pathology evaluations.

### Treatment approach

Patients received WLRT or VC-RT, delivered using either 2D/3DCRT or IMRT (18). Patients were immobilized in the supine using a thermoplastic head-and-neck mask and underwent CT-simulation with 3-5-mm slices. After 2010, Daily Cone-beam CT (CBCT) with soft tissue matching was performed for the first five fractions, followed by weekly CBCT for IMRT setup verification.

For cTis–T1 tumors, commonly used RT regimens included 63 Gy in 28 fractions (5.6 weeks), 51 Gy in 20 fractions, or 66 Gy in 33 fractions (5.5–6.6 weeks), delivered at five to six fractions per week. For cT2 tumors, total doses ranged from 60 Gy in 25 fractions (5 weeks) to 65.25 Gy in 29 fractions (5.8 weeks) or 70 Gy in 35 fractions (6 weeks).

### Target volume definition

For VC-RT, the gross tumor volume (GTV) was defined based on findings from fiberoptic laryngoscopy and available imaging. The high-risk clinical target volume (CTV-HR) was generated by an isotropic 5-mm expansion of the GTV. The low-risk clinical target volume (CTV-LR) consisted of a 1-cm isotropic expansion of the GTV, edited to respect anatomic boundaries and exclude soft tissues outside the thyroid cartilage, the contralateral vocal cord (if anterior commissure was uninvolved), the esophagus, and air cavities. The planning target volume (PTV) was generated by expanding the CTV by 5mm in all directions, with a 1-cm margin in the superior–inferior axis to account for setup uncertainties and laryngeal motion.

For patients treated with conventional 2D-CRT for WLRT, treatment fields were defined using bony landmarks. The superior border was typically placed at the mid-hyoid bone, the inferior border at the caudal edge of the first tracheal ring for cT1 and the second tracheal ring for cT2 disease, the anterior border extended to include the thyroid cartilage with a small skin margin, and the posterior border was positioned at the anterior aspect of the vertebral bodies to adequately cover the larynx while minimizing dose to the spinal cord.

For patients treated with 3DCRT/IMRT for WLRT, the GTV was defined based on fiberoptic laryngoscopy and available imaging. The CTV included the involved vocal cord(s) with a 5–10-mm margin to encompass potential microscopic disease, adjusted to respect anatomic boundaries. The CTV was expanded to include the entire thyroid cartilage while excluding the esophagus and air cavities. PTV was generated by a 5-mm isotropic expansion of the CTV in all directions to account for setup uncertainties and laryngeal motion.

### Post-treatment evaluation and follow-up

Patients underwent fiberoptic nasopharyngolaryngoscopy every 3 months during the first 2 years, every 4 months in the third year, every 6 months during years 4 and 5, and annually thereafter. Post-RT imaging was obtained as clinically indicated. For the purposes of the primary endpoint, tracheostomy was classified as treatment-related when performed after completion of RT in the setting of airway compromise without evidence of recurrent or persistent disease. Exclusion of recurrence was based on a combination of (1) fiberoptic nasopharyngolaryngoscopy at the time of or preceding the tracheostomy decision, (2) CT imaging of the neck with contrast, and (3) biopsy of any suspicious mucosal lesion where clinically indicated. In all 20 cases classified as edema-related tracheostomy, recurrence was excluded by at least two of the above modalities. No case in this cohort was deemed ambiguous with concurrent edema and suspected recurrence.

### Statistical methods

TR was defined as tracheostomy performed ≥6 months after radiotherapy for treatment-related airway compromise in the absence of recurrence. TR and local failure (LF) were estimated using the cumulative incidence method, with death treated as a competing risk. OS was analyzed using the Kaplan–Meier method and compared using the log-rank test. Time-to-event outcomes were calculated from the date of diagnosis to the first event. All reported p-values were two-sided, with statistical significance defined as p < 0.05. Odds ratios (OS) with 95% confidence intervals (CIs) were reported when applicable. Analyses were conducted using SAS version 9.4 (SAS Institute Inc., Cary, NC), and figures were generated using GraphPad Prism 7.

## Results

### Patient, tumor, and treatment characteristics

A total of 247 patients were included. The median age was 59 years (range, 30–92). Most patients were men (97.6%) and smokers (78.9%), whereas 4.5% reported alcohol consumption. T1a disease was present in 81.0% of patients. WLRT was delivered to 67.2% and VC-RT to 32.8%. Overall, 41.7% of patients received IMRT (**Table 1**).

**Table 1:** Patient, tumor, and treatment characteristics.

Characteristic	Total (n=247)	WLRT (n=166)	VC-RT (n=81)	P value
Follow-up, median (range), (months)	38.2 (1.61-192)	42.1 (1.6-194)	43.4 (1.6-144)	0.21
Age median (range), (years)	59 (30–92)	50 (30-92)	57 (32-90)	0.19
Gender				
Female	6 (2.4%)	3 (1.8%)	3 (3.7%)	0.397
Male	241 (97.6%)	163 (98.2%)	78 (96.3%)	
Performance status (ECOG)				
0	210 (85%)	150 (90.4%)	60 (74.1%)	**0.002**
1	34 (13.8%)	15 (9%)	19 (23.4%)	
2	3 (1.2%)	1 (0.06%)	2 (2.5%)	
Smoking storyhistory				
No	52 (21.1%)	29 (17.5%)	23 (28.4%)	**0.048**
Yes	195 (78.9%)	137 (82.5%)	58 (71.6%)	
Alcohol use				
No	236 (95.5%)	159 (95.8%)	77 (95.1%)	0.754
Yes	11 (4.5%)	7 (4.2%)	4 (4.9%)	
cT- category				
Tis	3 (1.2%)	3 (1.8%)	0	**<0.001**
T1a	200 (81.0%)	137 (82.5%)	63 (77.8%)	
T1b	17 (6.9%)	17 (10.2%)	0	
T2	27 (10.9%)	9 (5.4%)	18 (22.2%)	
Overall stage group				
Stage 0	3 (1.2%)	3 (1.8%)	0	**<0.001**
Stage I	217 (87.9%)	154 (92.8%)	63 (77.8%)	
Stage II	27 (10.9%)	9 (5.4%)	18 (22.2%)	
Radiotherapy technique				
2D RT				
3DCRT	144 (58.3%)	119 (71.7%)	25 (30.9%)	**<0.001**
IMRT	103 (41.7%)	47 (28.3%)	56 (69.1%)	
Fractionation				
Conventional				
66Gy/33fraction/5 fractions/week	29 (80.6%)	4 (57.1%)	25 (86.2%)	**0.001**
70Gy/35frcations/5 fractions/week	7 (19.4%)	3 (42.9%)	4 (13.8%)	
Accelerated conventional				
66Gy/33fraction/6 fractions/week	26 (89.7%)	17 (89.5%)	9 (90.0%)	
70Gy/35frcations/6 fractions/week	3 (10.3%)	2 (10.5%)	1 (10.0%)	
Accelerated hypofractionation				
51Gy/20fractions/5fraction/week	2 (1.2%)	2 (5.0%)	0	
60Gy/25fractions/5fractions/week	8 (4.9%)	1 (2.5%)	7 (5.7%)	
63Gy/28fractions/5 fractions/week	138 (85.2%)	26 (65.0%)	112 (91.8%)	
65.25Gy/29 fractions/5 fractions/week	14 (8.6%)	11 (27.5%)	3 (2.5%)	
Tracheostomy, irrespective of the underlying cause				
No	211 (85.4%)	132 (79.5%)	79 (97.5%)	**0.001**
Yes	36 (14.6%)	34 (20.5%)	2 (2.5%)	
Tracheostomy, based on the underlying cause				
Edema	20 (100%)	20 (100%)	0	**0.001**
Recurrence	211 (85.4%)	132 (79.5%)	79 (97.5%)	
Salvage laryngectomy				
No	227 (91.9%)	150 (90.4%)	77 (95.1%)	0.23
Yes	20 (8.1%)	16 (16.6%)	4 (4.9%)	

Bold values denote statistical significance, with a p value <0.05.

### Tracheostomy rate

**Figure 1:**
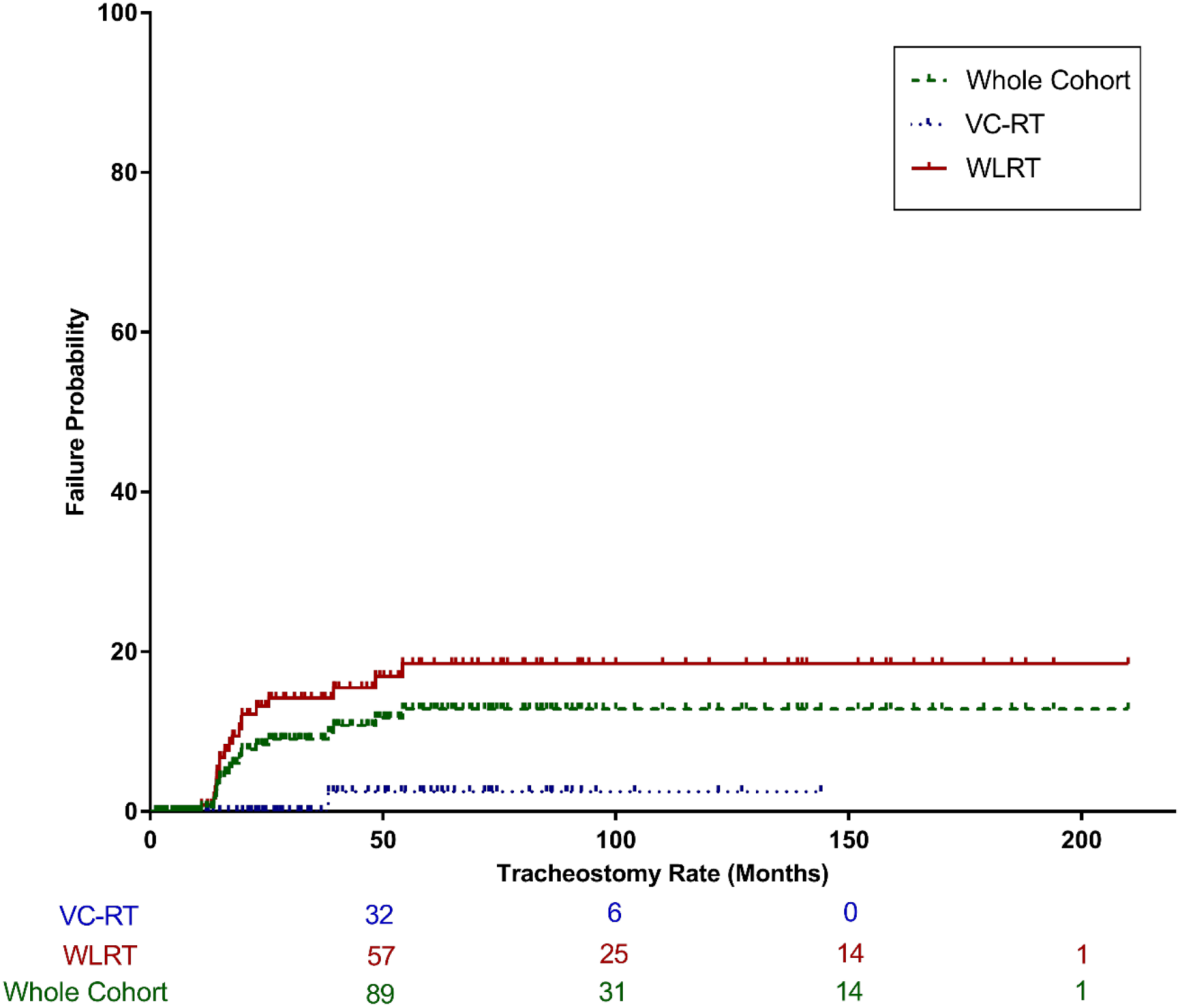
Cumulative incidence of tracheostomy for the entire cohort and by treatment group (VC-RT vs. WLRT). VC-RT, vocal cord only radiotherapy; WLRT, whole larynx radiotherapy.

Overall, 36 of 247 patients (14.6%) required tracheostomy for any reason. Of these, 20 (8.1% of the total cohort) underwent tracheostomy due to radiation-related edema in the absence of recurrence, constituting the primary endpoint. Tracheostomy was reversed in 8 of 20 patients (40%) after a median of 13.4 months. The 3-year TR for the entire cohort was 9.7% (95% CI, 5.28%–13.75%) and was significantly lower with VC-RT than with WLRT [0% (95% CI, NA%–NA%) vs. 14.22% (95% CI, 8.39-21,27%); p < 0.0001] (**Figure 1**). Multivariate analysis revealed no independent predictors of TR.

### Local failure

**Figure 2:**
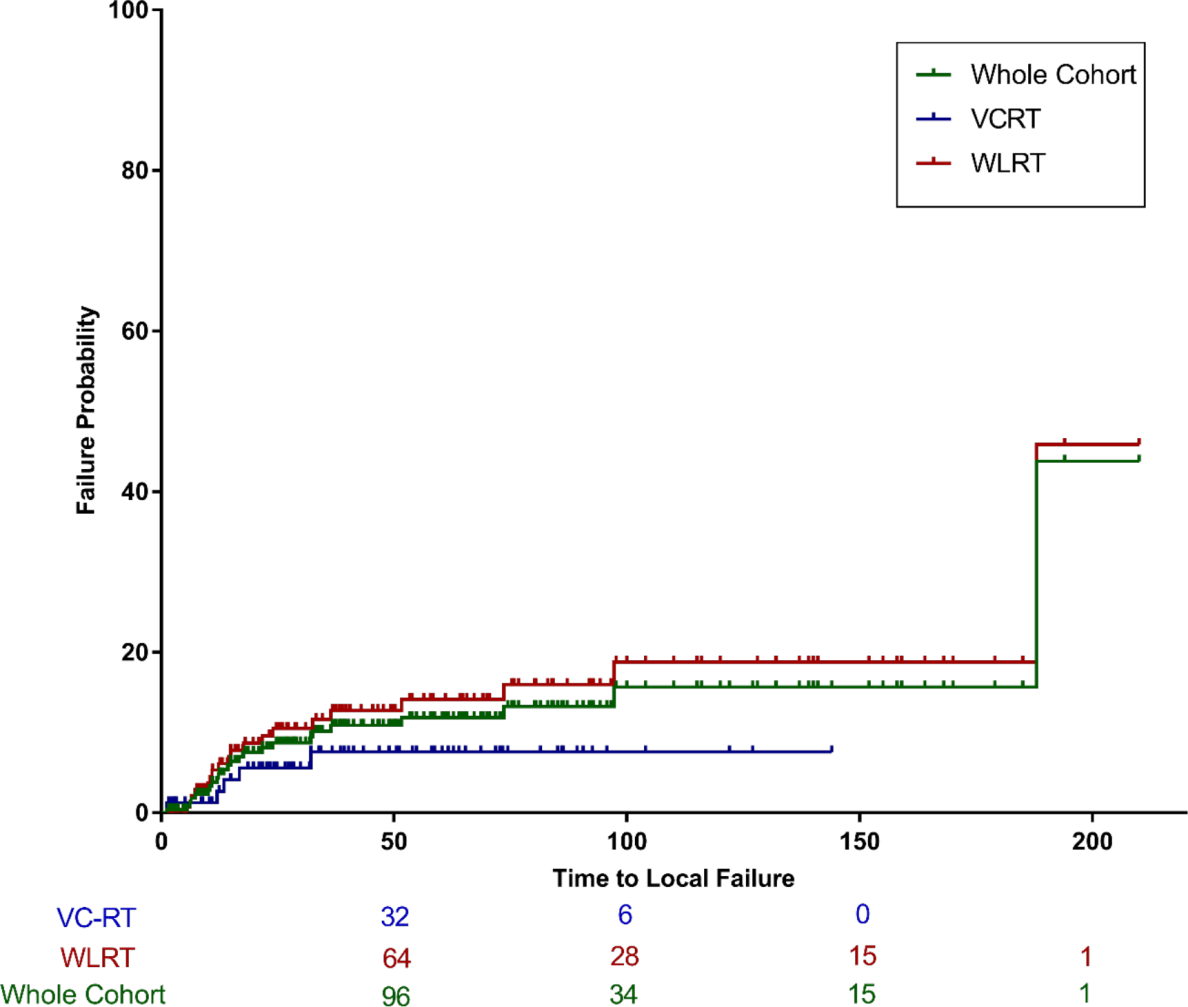
Cumulative incidence of local failure for the entire cohort and by treatment group (VC-RT vs. WLRT). VC-RT, vocal cord only radiotherapy; WLRT, whole larynx radiotherapy.

Local failure occurred in 24 patients (9.7%), with 20 patients (8.1%) undergoing salvage laryngectomy. For the whole cohort, the 3-year cumulative incidence of LF was 10.1% (95% CI, 6.3–15.1%). The 3-year cumulative incidence of LF was 7.6% (95% CI, 2.7–15.8%) for VC-RT, and 11.6% (95% CI, 6.6–18.2%) for WLRT (*p*=0.183) (**Figure 2**).

### Overall survival

**Figure 3:**
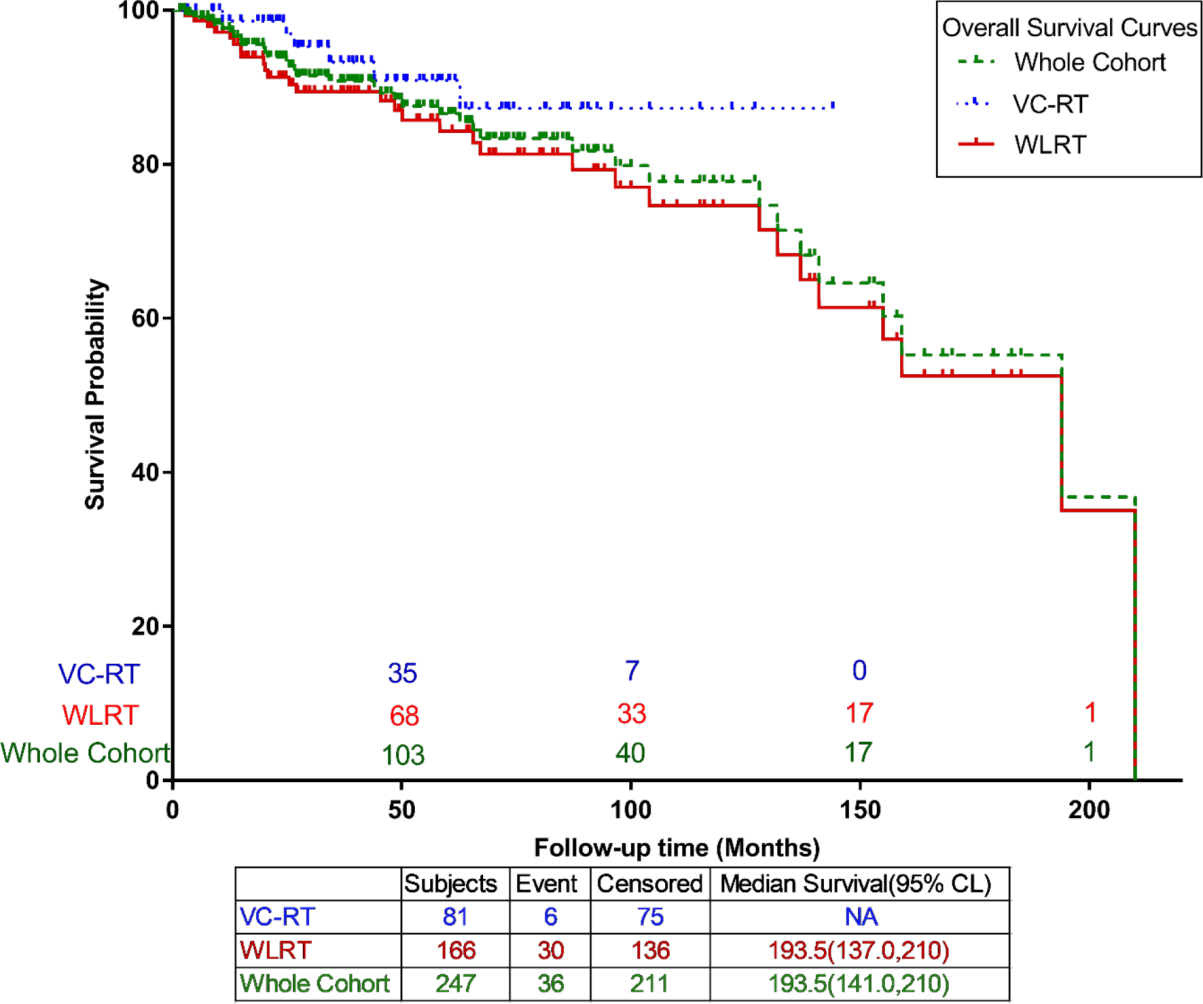
Kaplan–Meier curve for the entire cohort and by treatment group (VC-RT vs. WLRT). VC-RT, vocal cord only radiotherapy; WLRT, whole larynx radiotherapy.

At a median follow-up of 38.2 months, 36 deaths (14.6%) occurred. The 3-year OS for the entire cohort was 90.8% (95% CI, 86.2%–94.6%). The 3-year OS was 93.3% (95% CI, 85.5%–98.2%) for VC-RT and 89.4% (95% CI, 83.3%–94.2%) for WLRT (*p*=0.16) (**Figure 3**).

### Toxicity

Most acute skin toxicities were grade I–II. Aspiration pneumonia occurred in five patients (2.0%).

## Discussion

RT remains a cornerstone in the management of ESGC (19), with modern RT techniques enabling improved target conformity and reduced toxicity (20). In this multi-institutional study, VC-RT was associated with a significantly lower rate of tracheostomy while maintaining excellent oncologic outcomes. The 3-year TR was markedly lower with VC-RT compared with WLRT, whereas LC and OS remained comparable despite a higher proportion of cT2 disease in the VC-RT cohort. These findings support the oncologic adequacy of the VC-RT approach in appropriately selected patients (21).

Consistent with prior literature, both WLRT and VC-RT achieved high rates of LC and OS in ESGC (22, 23). Historical WLRT series report 5-year LC rates of 85%–95% for cT1 and 70%–85% for cT2 disease, which formed the basis for WLRT in the treatment of ESGC (22). More recent studies using modern imaging and image-guided techniques demonstrate that VC-RT can achieve comparable oncologic outcomes in carefully selected patients, with reported 3- to 5-year LC rates of approximately 88%–95% (23–25). In our cohort, 3-year LC was similar between VC-RT and WLRT (7.6% vs. 11.6%, p=0.151), despite a higher proportion of cT2 disease in the VC-RT group, supporting the oncologic safety of focal approaches when delivered with modern radiotherapy techniques.

OS in ESGC is excellent, reflecting the curable nature of cTis–T2 disease, with published 3-year rates ranging from 85% to 95% (26–28). In our study, the 3-year OS was 93.3% (95% CI, 85.5%–98.2%) for VC-RT and 89.4% (95% CI, 83.3%–94.2%) for WLRT (*p*=0.16), with comparable outcomes despite more advanced disease in the VC-RT cohort. These findings further support the oncologic adequacy of a focal, organ-preserving approach in appropriately selected patients.

Tracheostomy following definitive RT for ESGC is uncommon but represents a clinically meaningful complication associated with laryngeal edema, fibrosis, or airway compromise (29). Historical WLRT series report tracheostomy rates ranging from 2% to 10%, whereas more conformal approaches, such as VC-RT, are associated with lower rates due to reduced dose to uninvolved laryngeal structures (30). In our multi-institutional cohort, tracheostomy occurred in 20.5% of WLRT patients versus 0% with VC-RT (p<0.001), with edema-related events observed exclusively in the WLRT group. Although dosimetric data were not systematically collected in our cohort, these findings are consistent with established dose–volume relationships, where higher mean laryngeal dose increases the risk of edema without improving LC. A recent meta-analysis of 7,033 patients reported a pooled dysfunctional larynx incidence of only 0.3%—the concentration of these events in our WLRT is consistent with known dose–volume relationships for laryngeal edema (21). Sanguineti et al. demonstrated that mean laryngeal dose is the strongest independent predictor of moderate-to-severe edema, with risk rising sharply above 44 Gy (31), and NTCP modeling confirmed a clear volume effect with a TD50 of approximately 47 Gy (32). Inoue et al. showed directly in a randomized comparison of field sizes in cT1 glottic cancer that larger fields produced five times more persistent edema (21% vs. 4%, p<0.02) without any improvement in LC (33). While recurrence itself drives airway obstruction, the high prevalence of edema-related tracheostomy in our WLRT cohort implicates treatment-related toxicity from bilateral laryngeal exposure—including radiation-induced mucositis, fibrosis, and microvascular compromise rather than disease biology alone. Together, our results support treatment de-escalation with VC-RT as a strategy to reduce airway morbidity while maintaining excellent oncologic outcomes and toxicity (34).

Dosimetric studies show that VC-RT significantly reduces carotid dose compared with WLRT, without increasing contralateral vocal cord failure (35), and that some radiation oncologists may have (36). This reduction in dose to uninvolved structures may decrease the risk of carotid stenosis and cerebrovascular events while maintaining adequate target coverage and LC (37, 38). IMRT further reduces the mean carotid dose from 38 Gy with opposed lateral fields to as low as 4 Gy with VC-RT and decreases the carotid V50Gy from 77% to 0% (39). In our cohort, although VC-RT was associated with improved functional outcomes and lower TR, dosimetric data were not systematically collected, representing a limitation of this study. Another key limitation is the strong correlation between treatment volume and radiotherapy technique, reflecting the sequential institutional adoption of IMRT and VC-RT over the study period: WLRT was predominantly delivered with 3DCRT during the earlier era, whereas VC-RT was adopted alongside IMRT as the institutional standard evolved in line with NCCN guidelines. This collinearity limits our ability to fully isolate the independent contribution of target volume reduction from that of technical advancement. This pattern, however, mirrors real-world practice evolution in the field. Prospective studies with standardized techniques across both target volume approaches are needed to definitively address this question. Adoption of VC-RT remains limited by the lack of consensus on target delineation, and further prospective studies are needed to define its vascular and functional benefits (40).

The principles of focal therapy demonstrated in our study provide a strong rationale for the future development of laryngeal stereotactic body radiotherapy (SBRT). Mature phase II data from the glottic SBRT trial reported by David J. Sher show encouraging local control, with 1- and 2-year local failure rates of 4% and 8%, respectively, alongside acceptable toxicity and markedly shortened treatment duration (41). Similarly, ultra-hypofractionated regimens described by Giovanni Sanguineti demonstrate excellent tumor control but highlight a non-negligible risk of late necrosis, reinforcing the narrow therapeutic window of SBRT (42). As emphasized by Jean Le Guévelou, future progress will depend on careful patient selection, risk-adapted fractionation, and advanced image guidance to minimize toxicity (43). Prospective comparative studies against VC-RT and conventional RT, with strong emphasis on functional outcomes such as voice quality, are needed to define the role of SBRT and support its safe integration into clinical practice (44).

This study reflects real-world practice and highlights tracheostomy as a meaningful functional endpoint. Although limited by its retrospective design, treatment of heterogeneity, and lack of detailed dosimetric and voice outcome data, our findings support treatment of de-escalation with VC-RT and warrant prospective validation.

## Conclusion

In this multi-institutional retrospective study, VC-RT was associated with a significantly lower rate of treatment-related tracheostomy compared with WLRT, without evidence of inferior local control or overall survival. These findings support further prospective evaluation of VC-RT as a function-preserving strategy in appropriately selected patients with early-stage glottic cancer.

## Data Availability

The raw data supporting the conclusions of this article will be made available by the authors, without undue reservation.
